# LOW-INTENSITY PEER-LED EXERCISE DOES NOT CHANGE PHYSICAL ACTIVITY OR BALANCE IN OLDER ADULTS

**DOI:** 10.1093/geroni/igae098.4236

**Published:** 2024-12-31

**Authors:** Chitra Banarjee, Jethro Raphael Suarez, Renoa Choudhury, Joon-Hyuk Park, Rui Xie, Jeffrey Stout, Ladda Thiamwong

**Affiliations:** University of Central Florida, Orlando, Florida, United States; University of Central Florida; Virginia Polytechnic Institute and State University, Blacksburg, Virginia, United States; University of Central Florida, Orlando, Florida, United States; University of Central Florida, Orlando, Florida, United States; University of Central Florida, Orlando, Florida, United States; University of Central Florida, Orlando, Florida, United States

## Abstract

Decreased physical activity in aging adults is associated with increased fear of falling (FOF) and decreased static balance. These results varied by the level of engaged physical activity (i.e., light, moderate, vigorous). This study examined the effect of a low-intensity peer-led intervention on step count, FOF, and static balance. We recruited 65 community-dwelling older adults (mean age = 73.52 years ± 5.66; 87.7% female) at two-time points (mean interval = 177.66 days ± 80.33) to evaluate longitudinal changes in step count, perceived fear of falling (Short Falls Efficacy Scale-International (FES-I)), and objective static balance (BTrackS Balance System (BBS)). Step count was recorded over a 7-day interval at each time point. For FOF, participants completed the short FES-I, a 7-item self-report questionnaire. For static balance, participants stood on a balance plate for 3 20-second trials that were averaged to determine the center of pressure path length. Forty-one participants attended a once-weekly peer-led exercise for one hour. Wilcoxon tests revealed no significant effect of this intervention in step count (p=0.984), FOF (p=0.1972), or static balance (p=0.8136). This suggests once-weekly low-intensity exercise did not significantly affect physical activity and balance measures. Limitations include a predominantly female sample and the low-intensity nature of the intervention. Further research should investigate the effects of a longer duration, higher-intensity peer-led exercises in a larger, more diverse sample.

